# Supplementary material for: CARD8 inflammasome activation during HIV-1 cell-to-cell transmission
Source: bioRxiv. 2025 Feb 15:2024.08.21.608981. Originally published 2024 Aug 22. Preprint. [Version 2] doi: 10.1101/2024.08.21.608981 (PMC11370340; doi:10.1101/2024.08.21.608981)
Supplement: 1 [file NIHPP2024.08.21.608981V2-supplement-1.pdf]

bioRxiv preprint doi: https://doi.org/10.1101/2024.08.21.608981; this version posted February 15, 2025. The copyright holder for this preprint (which was not certified by peer review) is the author/funder, who has granted bioRxiv a license to display the preprint in perpetuity. It is made available under aCC-BY 4.0 International license.

Table S1: Protease inhibitor resistance mutations and relative CARD8 cleavage.

Clone name	Reported PI-resistance mutations in HIV^{PR}	HIV^{gag} mutation	Strongest PI-R[1]	% CARD8 cleavage (relative to HIV$_{LAI}$)	Additional amino acid changes in HIV^{PR} relative to NL4.3
HIV-1$_{LAI}$	wildtype	wildtype	wildtype	100	37S
CA126802 (PI-R1)	11I, 32I, 33F, 46I, 47V, 54M, 58E, 73S, 84V, 89V, 90M	431V (NC/p1) 437N (NC/p1) 453LF (p1/p6)	FPV, LPV, TPV, DRV	51	10I, 12K, 13V, 20V, 35G, 36I, 37D, 57K, 63P, 64V, 66V, 7IV
**CA122805 (PI-R2)**	**10F, 33F, 43T, 46L, 54V, 82A, 84V, 90M**	**431V (NC/p1) 532S (p1/p6)**	**SQV**	**7**	16A,19I, 20R, 35D, 36L, 37D, 55R, 57K, 60E, 62V, 63P, 71V, 93L
CA126805 (PI-R3)	33F, 43T, 46I, 48V, 50V, 54S, 82A	437N (NC/p1) 449VF (p1/p6) PTAP insertion (p6)	SQV, IDV, LPV	**8**	not determined*
CA96457 (PI-R5)	48V, 53L, 54V, 82A, 90M	436R (NC/p1)	SQV	11	10I, 37D, 63P, 71T, 77I, 93L
**CA96458 (PI-R9)**	**10F, 30N, 33F, 43T, 84V, 88D, 90M**	**431V (NC/p1)**	**NFV, SQV**	**24**	15V, 35D, 36L, 37E, 60E, 62V, 63P
CA50834-1 (PI-R10)	24I, 46L, 54V, 76V, 82A	431V (NC/p1)	IDV, LPV	38	10I, 14R, 35D, 36I, 37E, 63P,71V
**CA96451 (PI-R12)**	**32I, 33F, 43T, 46I, 47V, 54M, 73S, 82A, 89V, 90M**	**437N (NC/p1) PTAP insertion (p6)**	**FPV, LPV**	**192**	10V, 12V, 13V, 15V, 20M, 60E, 61N, 62V, 63P, 67Y, 69K, 71I, 72L, 77I
**CA20392-1 (PI-R13)**	**24I, 46L, 54V, 82A**	**431V (NC/p1)**	**LPV**	**206**	10I, 14R, 35D, 36I, 37E, 63P, 71V

Table S1 shows the protease inhibitor-resistant (PI-R) clones assayed in **Figure 5** with corresponding mutations in HIV protease (HIV^{PR}) and HIV^{gag}. [1]These clones were previously cloned and assayed for PI-R in (Varghese et al., 2013). The PI-R subset used in Figure 5B are bolded and highlighted in red or green and denote either hypo- or hyper-active CARD8 cleavage, respectively. The last column reports additional amino acid changes in the PI-R clones that were observed via whole plasmid Oxford Nanopore sequencing. *We were unable to sequence verify PI-R3 due to poor plasmid quality. NFV-nelfinavir; FPV-fosamprenavir; SQV- saquinavir; IDV- indinavir; LPV- lopinavir; TPV- tipranavir; DRV- darunavir. The consensus subtype B sequence can be found on the Stanford HIV Drug Resistance Database (HIVDB) ("Stanford - HIV Drug Resistance Database," n.d.). Relative CARD8 cleavage was determined by quantifying band volume of the CARD8 cleavage product in BioRad Image Lab 6 and comparing to cleavage with HIV-1$_{LAI}$.

bioRxiv preprint doi: https://doi.org/10.1101/2024.08.21.608981; this version posted February 15, 2025. The copyright holder for this preprint (which was not certified by peer review) is the author/funder, who has granted bioRxiv a license to display the preprint in perpetuity. It is made available under aCC-BY 4.0 International license.

Table S2:

sgRNA	Sequence
CARD8 sgRNA1	CUCUGCAGUGACAUCAAACA
CARD8 sgRNA2	UGACGAUUGCGUUUGGUUCC
CARD8 sgRNA3	AGCGUUUGGUUCCCCACUGC
AAVS1 sgRNA 1	GUUAAUGUGGCUCUGGUUCU
AAVS1 sgRNA 2	ACCCCACAGUGGGGCCACUA
AAVS1 sgRNA 3	CCUUCCUAGUCUCCUGAUAU
NLRP3 sgRNA 1	GCUCAGAAUGCUCAUCAUCG
NLRP3 sgRNA 2	GAUGAUGUUGGACUGGGCAU
NLRP3 sgRNA 3	CAAGGCUCACCUCCCGACAG

CD4+ T cell isolation, infection, and coculture

Primary CD4+ T cells were isolated via positive selection using the EasySep™ Release Human CD4 Positive selection kit (Stem Cell Technologies Cat: 17752) according to the manufacturer's instructions from PBMCs collected from blood donors and seeded at 2.5x10^6 cells/mL in the presence of 100U/mL IL-2. T cells were activated 24 hours post-isolation with CD3/CD28 activation beads (Miltenyi Biotech Cat: 130-091-441). Activation beads were removed according to the manufacturer's protocols 24 hours later for infection. For infection, T cells were suspended at 1-1.5x10^6 cells/mL in 15mL conical tubes containing 8μg/mL polybrene, 100U/mL IL-2, and HIV-$1_{NL4.3\text{-}BaL}$ then spinoculated at 1100g at 30°C for 90 min. Three days post-infection, CD4s were assessed for intracellular $p24^{gag}$ via flow cytometry (~10% infected) then washed thrice with PBS before coculturing with MDMs. CD4:MDM coculture RPMI media was supplemented with 100U/mL IL-2, 20ng/mL GM-CSF, 20ng/mL M-CSF, and 500ng/mL Pam3CSK4.

bioRxiv preprint doi: https://doi.org/10.1101/2024.08.21.608981; this version posted February 15, 2025. The copyright holder for this preprint (which was not certified by peer review) is the author/funder, who has granted bioRxiv a license to display the preprint in perpetuity. It is made available under aCC-BY 4.0 International license.

Supplemental Note

There have been 4 different sublines of THP-1 cells previously characterized (Kasai et al., 2022). Using short tandem repeat (STR) profiling, we were able to distinguish the WT THP-1 cell stocks that were used as the parental line for knockouts and complemented knockouts in this work and our prior work (Kulsuptrakul et al., 2023), as distinct from WT THP-1 cells sourced from ATCC at 3 different loci (**Figure A1A**). Of note, unlike the THP-1 cells used here (referred to as JK THP-1), ATCC THP-1 cells elicited IL-1β secretion in the absence of HIV-1 infection in the presence of 20μg/mL DEAE-dextran (**Figure A1B**). Unless otherwise specified, any mention of "THP-1 cells" are referring to our JK THP-1 cells, not ATCC THP-1 cells. Nonetheless, given the sensitivity of some THP-1 sublines to elicit an inflammasome response in the presence of DEAE-dextran, we assessed whether or not we could establish systems to measure HIV-1 induced CARD8-dependent inflammasome activation in the absence of DEAE-dextran. Thus, we infected either wildtype (WT) or *CARD8* knockout (KO) THP-1 cells with wildtype HIV-1_{LAI} in either the presence or absence of DEAE-dextran and measured cell death and IL-1β secretion 24 hours post-infection as readouts of inflammasome activation. We found that despite achieving similar levels of infection (20-30%) as measured by intracellular $p24^{gag}$ after spinoculation with and without DEAE-dextran (**Figure A1C,** left), we detected robust CARD8-dependent inflammasome activation in WT THP-1 cells infected only in the presence of DEAE-dextran (**Figure A1C,** middle and right). These data suggest that cationic polymer is necessary to observe HIV-dependent CARD8 inflammasome activation in our cell-free system.

bioRxiv preprint doi: https://doi.org/10.1101/2024.08.21.608981; this version posted February 15, 2025. The copyright holder for this preprint (which was not certified by peer review) is the author/funder, who has granted bioRxiv a license to display the preprint in perpetuity. It is made available under aCC-BY 4.0 International license.

A

Cell Line:	JK THP-1		ATCC THP-1	
Expasy Best Match Cell Line No:	CVCL_5115		CVCL_E2QQ	
Marker	Allele 1	Allele 2	Allele 1	Allele 2
AMEL	X	Y	X	Y
D3S1358	15	17	15	17
D1S1656	16	17	16	17
D2S441	10	11	10	11
D10S1248	14		14	
**D13S317**	8	13	13	
Penta E	11	15	11	15
D16S539	11	12	11	12
D18S51	13	14	13	14
D2S1338	17	18	17	18
CSF1PO	11	13	11	13
Penta D	10	12	10	12
TH01	8	9.3	8	9.3
**vWA**	16	17	16	
D21S11	30	31.2	30	31.2
D7S820	10		10	
D5S818	11	12	11	12
TPOX	8	11	8	11
DYS391	10		10	
D8S1179	10	14	10	14
**D12S391**	18	19	19	
D19S433	12.2	13	12.2	13
FGA	24	25	24	25
D22S1045	11	15	11	15

B

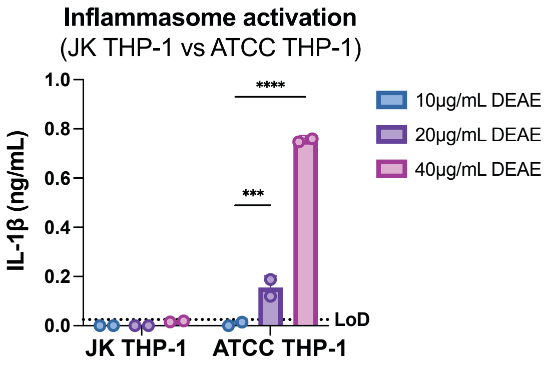

C

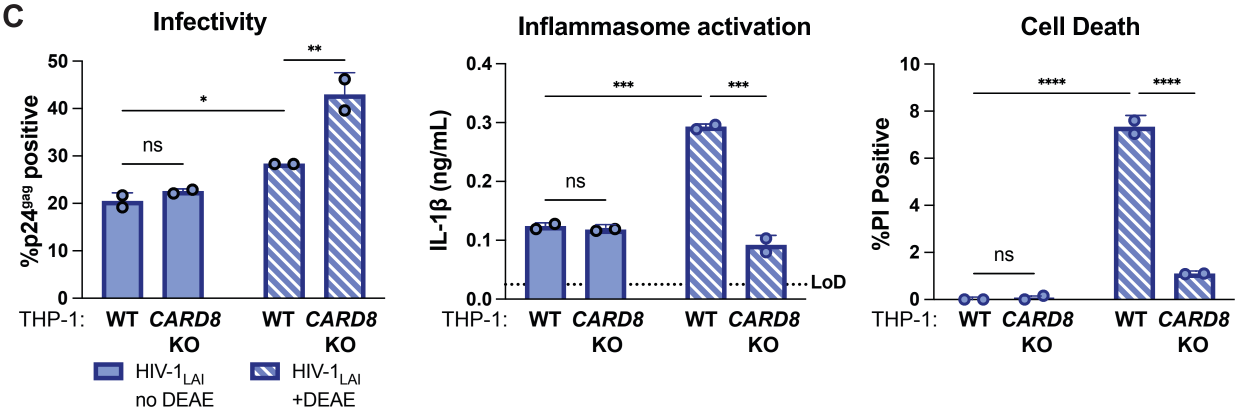

Figure A1. Characterization of THP-1 cells. (A) Promega GenePrint® 24 system STR analysis summary of our JK THP-1 cells versus ATCC THP-1 cells. Cell line authentication was done by TransnetYX, Inc. by following the protocol described in ANSI/ATCC ASN-0002-2011. The STR alleles were searched on the ATCC Database and the Expasy best match cell numbers for each cell line had a 100% database match. Distinguishing loci are highlighted in yellow and distinguishing alleles are in red. **(B)** JK and ATCC THP-1 cells were primed with Pam3CSK4 overnight then treated with increasing doses of DEAE-dextran for 24 hours before probing for IL-1β secretion. (**C**) Wildtype (WT) or *CARD8* knockout (KO) THP-1 cells were infected with wildtype HIV-1_{LAI} at the same MOI in the presence or absence of DEAE-dextran (10µg/mL) then harvested after 24 hours and assayed for: left) percent infection via intracellular $p24^{gag}$, middle) inflammasome activation by IL-1β secretion via IL-1R reporter assay, and right) cell death via propidium iodide (PI) dye uptake using flow cytometry. %PI positive and IL-1 levels are normalized to mock control. Dotted line indicates limit of detection (LoD). Datasets represent mean ± SD (n=2 biological replicates). Two-way ANOVA with (B) Sidak's or (C) Tukey's test using GraphPad Prism 10. ns = not significant, *p<0.05,**p<0.01, ***p<0.001, ****p<0.0001.

bioRxiv preprint doi: https://doi.org/10.1101/2024.08.21.608981; this version posted February 15, 2025. The copyright holder for this preprint (which was not certified by peer review) is the author/funder, who has granted bioRxiv a license to display the preprint in perpetuity. It is made available under aCC-BY 4.0 International license.

Supplemental Note References

Kasai F, Hirayama N, Fukushima M, Kohara A, Nakamura Y. 2022. THP-1 reference data: Proposal of an in vitro branched evolution model for cancer cell lines. *Int J Cancer* **151**:463–472. doi:10.1002/ijc.34019

Kulsuptrakul J, Turcotte EA, Emerman M, Mitchell PS. 2023. A human-specific motif facilitates CARD8 inflammasome activation after HIV-1 infection. *eLife* **12**:e84108. doi:10.7554/eLife.84108
