# Supplementary material for: CARD8 inflammasome activation during HIV-1 cell-to-cell transmission
Source: bioRxiv. 2024 Aug 22:2024.08.21.608981. Preprint. [Version 1] doi: 10.1101/2024.08.21.608981 (PMC11370340; doi:10.1101/2024.08.21.608981)
Supplement: Supplementary file 1 [file NIHPP2024.08.21.608981v1-supplement-1.pdf]

bioRxiv preprint doi: https://doi.org/10.1101/2024.08.21.608981; this version posted August 22, 2024. The copyright holder for this preprint (which was not certified by peer review) is the author/funder, who has granted bioRxiv a license to display the preprint in perpetuity. It is made available under aCC-BY 4.0 International license.

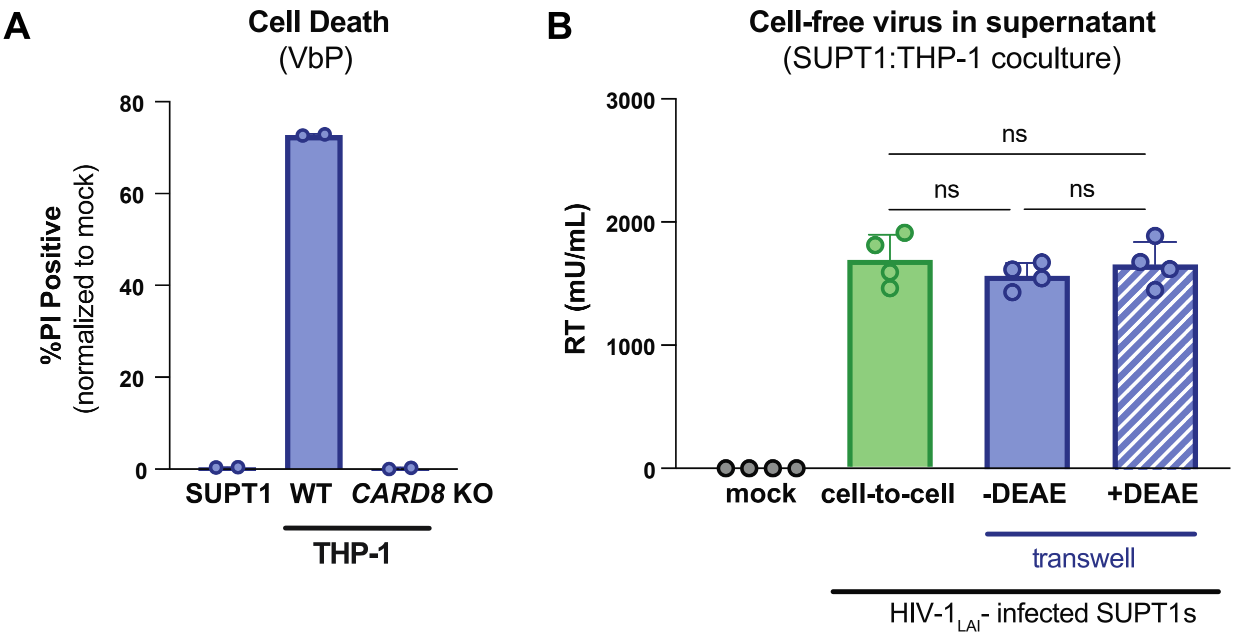

Figure 1- figure supplement 1. HIV-1 cell-to-cell infection induces inflammasome activation. (A) SUPT1 or THP-1 cells were primed with Pam3CSK4 (500ng/mL) overnight then treated with 5μM ValboroPro (VbP) for 24 hours then assessed for cell death via propidium iodide (PI) uptake. (B) SUPT1 cells were cocultured with THP-1 cells as in Figure 1D and supernatant in the cell-to-cell condition and in the supernatant outside of the transwell was sampled and measured for cell-free infectious HIV virions via reverse transcriptase (RT) assay. Datasets represent mean ± SD (A: n=2; B: n=4 biological replicates). B: Two-way ANOVA with Tukey's test using GraphPad Prism 10. ns = not significant

bioRxiv preprint doi: https://doi.org/10.1101/2024.08.21.608981; this version posted August 22, 2024. The copyright holder for this preprint (which was not certified by peer review) is the author/funder, who has granted bioRxiv a license to display the preprint in perpetuity. It is made available under aCC-BY 4.0 International license.

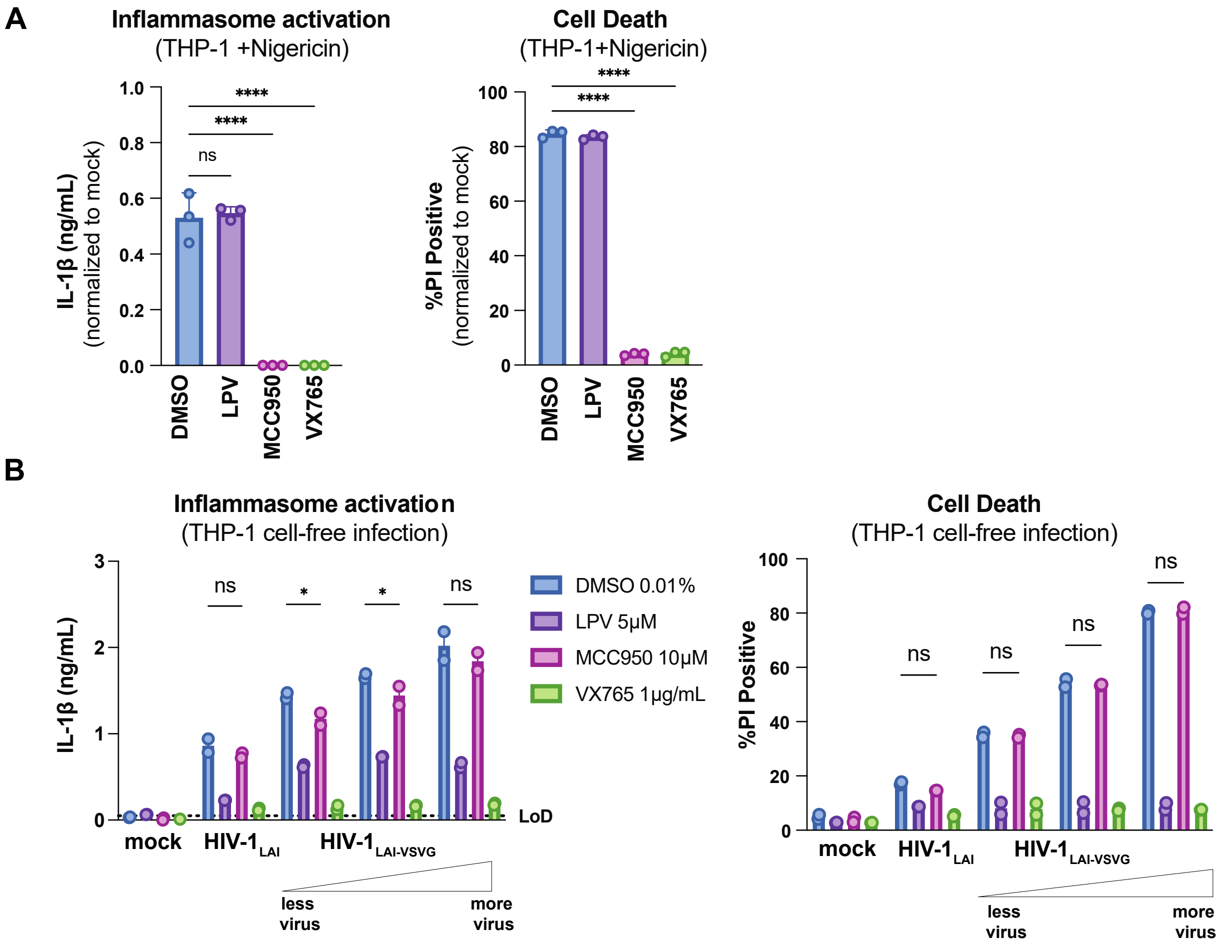

Figure 2- figure supplement 1. HIV-dependent inflammasome activation is largely NLRP3-independent. (**A**) Wildtype THP-1 cells were pre-treated with either DMSO, lopinavir (LPV), MCC950, or VX765 at 0.01%, 5µM, 10µM, or 1µg/mL, respectively, for 15 minutes prior to 4-hour treatment with 5µg/mL nigericin. Subsequent inflammasome activation was assessed via (A-left) IL-1β secretion via IL-1R reporter assay and (A-right) cell death via propidium iodide (PI) dye uptake. (**B**) Wildtype THP-1 cells were pre-treated with indicated inhibitors as in (A) then infected with either HIV-1$_{LAI}$ or increasing amounts of VSV-G pseudotyped HIV-1$_{LAI}$ (HIV-1$_{LAI-VSVG}$) in the presence of 10µg/mL DEAE-dextran. Subsequent inflammasome activation was assessed 24 hours post infection via IL-1β secretion and cell death as in (A). Dotted line indicates limit of detection (LoD). Datasets represent mean ± SD (A: n=3, B: n=2 biological replicates). One-way (A) or Two-way (B) ANOVA with Dunnett's test using GraphPad Prism 10. ns = not significant, *p<0.05,**p<0.01, ***p<0.001, ****p<0.0001.

bioRxiv preprint doi: https://doi.org/10.1101/2024.08.21.608981; this version posted August 22, 2024. The copyright holder for this preprint (which was not certified by peer review) is the author/funder, who has granted bioRxiv a license to display the preprint in perpetuity. It is made available under aCC-BY 4.0 International license.

357 **Table S1: Protease inhibitor resistance mutations and relative CARD8 cleavage.**

Clone name	PI-resistance mutations in HIV^{PR}	HIV^{gag} mutation	Strongest PI-R[1]	% CARD8 cleavage (relative to HIV_{LAI})
HIV-1_{LAI}	wildtype	wildtype	wildtype	100
CA126802 (PI-R1)	11I, 32I, 33F, 46I, 47V, 54M, 58E, 73S, 84V, 89V, 90M	A431V (NC/p1) I437N (NC/p1) P453LF (p1/p6)	FPV, LPV, TPV, DRV	51
**CA122805 (PI-R2)**	**10F, 33F, 43T, 46L, 54V, 82A, 84V, 90M**	**A431V (NC/p1) R532S (p1/p6)**	**SQV**	**7**
CA126805 (PI-R3)	33F, 43T, 46I, 48V, 50V, 54S, 82A	I437N (NC/p1) L449VF (p1/p6) PTAP insertion (p6)	SQV, IDV, LPV	**8**
CA96457 (PI-R5)	48V, 53L, 54V, 82A, 90M	K436R (NC/p1)	SQV	11
**CA96458 (PI-R9)**	**10F, 30N, 33F, 43T, 84V, 88D, 90M**	**A431V (NC/p1)**	**NFV, SQV**	**24**
CA50834-1 (PI-R10)	24I, 46I, 54V, 76V, 82T	A431V (NC/p1)	IDV, LPV	38
**CA96451 (PI-R12)**	**32I, 33F, 43T, 46I, 47V, 54M, 73S, 82A, 89V, 90M**	**I437N (NC/p1) PTAP insertion (p6)**	**FPV, LPV**	**192**
**CA20392-1 (PI-R13)**	**24I, 46L, 54V, 82A**	**A431V (NC/p1)**	**LPV**	**206**

bioRxiv preprint doi: https://doi.org/10.1101/2024.08.21.608981; this version posted August 22, 2024. The copyright holder for this preprint (which was not certified by peer review) is the author/funder, who has granted bioRxiv a license to display the preprint in perpetuity. It is made available under aCC-BY 4.0 International license.

Table S2:

sgRNA	Sequence
CARD8 sgRNA1	CUCUGCAGUGACAUCAAACA
CARD8 sgRNA2	UGACGAUUGCGUUUGGUUCC
CARD8 sgRNA3	AGCGUUUGGUUCCCCACUGC
AAVS1 sgRNA 1	GUUAAUGUGGCUCUGGUUCU
AAVS1 sgRNA 2	ACCCCACAGUGGGGCCACUA
AAVS1 sgRNA 3	CCUUCCUAGUCUCCUGAUAU
